# Inkjet-printed high-performance and mechanically flexible organic photodiodes for optical wireless communication

**DOI:** 10.1038/s41598-024-53796-5

**Published:** 2024-02-08

**Authors:** Luis Arturo Ruiz‐Preciado, Petr Pešek, Carlos Guerra-Yánez, Zabih Ghassemlooy, Stanislav Zvánovec, Gerardo Hernandez-Sosa

**Affiliations:** 1 Light Technology Institute, Karlsruhe Institute of Technology, Engesserstr. 13, 76131 Karlsruhe, Germany; 2 InnovationLab, Speyererstr. 4, 69115 Heidelberg, Germany; 3 Faculty of Electrical Engineering, Czech Technical University in Prague, Dejvice-Praha 6, 16627 Prague, Czech Republic; 4 Optical Communications Research Group, Faculty of Engineering and Environment, Northumbria University, Newcastle, UK; 5 Institute of Microstructure Technology, Karlsruhe Institute of Technology, Hermann-von-Helmholtz-Platz 1, 76344 Eggenstein-Leopoldshafen, Germany

**Keywords:** Electrical and electronic engineering, Materials for devices, Electronic devices, Polymers, Semiconductors, Imaging and sensing, Fibre optics and optical communications, Optoelectronic devices and components

## Abstract

Emerging areas such as the Internet of Things (IoT), wearable and wireless sensor networks require the implementation of optoelectronic devices that are cost-efficient, high-performing and capable of conforming to different surfaces. Organic semiconductors and their deposition via digital printing techniques have opened up new possibilities for optical devices that are particularly suitable for these innovative fields of application. In this work, we present the fabrication and characterization of high-performance organic photodiodes (OPDs) and their use as an optical receiver in an indoor visible light communication (VLC) system. We investigate and compare different device architectures including spin-coated, partially-printed, and fully-printed OPDs. The presented devices exhibited state-of-the-art performance and reached faster detection speeds than any other OPD previously reported as organic receivers in VLC systems. Finally, our results demonstrate that the high-performance of the fabricated OPDs can be maintained in the VLC system even after the fabrication method is transferred to a fully-inkjet-printed process deposited on a mechanically flexible substrate. A comparison between rigid and flexible samples shows absolute differences of only 0.2 b s^−1^ Hz^−1^ and 2.9 Mb s^−1^ for the spectral efficiency and the data rate, respectively.

## Introduction

Optical technologies have extensive applications in our daily lives and continue to offer multiple options for development in fields such as communication, healthcare, and automation^1,2^. In recent years, the relevance of optical technologies has become increasingly apparent thanks to emerging areas such as the internet of things (IoT), wearable and mobile devices. At the same time, the use of organic semiconductors has opened up new possibilities for optical devices that are particularly suitable for these innovative fields of application. In contrast to inorganic semiconductors, organic devices can be solution-processed, allowing for a wider range of substrates, offering light-weight and increased mechanical flexibility^3,4^. Moreover, organic semiconductors can be deposited via digital printing techniques, thus enabling higher freedom of design and additive manufacturing of integrated systems^5^. In this regard, inkjet printing has emerged as a particularly attractive approach, allowing high-throughput, personalization and cost-efficient production of electronic devices. In optical sensing, inkjet printing has been utilized to fabricate state-of-the-art organic photodiodes (OPDs) with comparable properties to their inorganic counterparts^6–10^. Due to the chemical nature of organic semiconductors, OPDs offer wide photoresponse tunability, making them suitable for a large range of applications requiring both broadband and wavelength-specific light detection^7,11–15^.

One of the main fields of application for optical technologies lies in the field of communications. In this regard, visible light communication (VLC), which uses the visible light band, has recently emerged as a promising complementary technology to the radio frequency (RF)-based wireless communication^16^. VLC, by its nature, offers different features including high data rates, not being affected by the RF-induced electromagnetic interference, and inherent security, thus making it a suitable technology for emerging applications such as virtual reality, indoor positioning, high-quality video streaming, smart lighting and Li-Fi^17^. Recent research work on VLC systems has achieved important progress by (i) adopting optimized techniques^18^; (ii) using innovative methods such as simultaneous power generation and data transfer^19,20^; and (iii) successfully demonstrating proof-of-concept applications^21–23^. The majority of previous studies, however, have been reported on conventional inorganic-based transmitters and receivers. Research works reported on organic-based devices are limited, with the focus being on organic transmitters such as organic light emitting diodes (OLEDs)^18,24–29^. Although OPDs have shown significant progress in terms of detection speed, with literature reports showing values that are well-suited for VLC applications^5,30,31^, there are still limited literature reports successfully demonstrating data transmission in VLC systems using OPD-receivers. Moreover, most of these reports utilize OPDs with low bandwidths and deposited mainly on rigid substrates^19,32–38^. These studies achieved spin-coated OPDs with bandwidths between 0.5 and 3 MHz for rigid samples and below 500 kHz for flexible samples.

In this work, we present the fabrication and characterization of high-performance OPDs and their successful integration in a VLC system. We investigate and compare different device architectures including spin-coated, partially-printed, and fully-printed OPDs. Our results demonstrate that the high-performance of the fabricated OPDs can be maintained in the VLC system even after the fabrication method is transferred to a fully-inkjet-printed process deposited on a mechanically flexible substrate. At the device level, we achieve responsivities above 300 mA W^−1^ and reach bandwidths within the range of 2 to 5 MHz at a reverse voltage of  − 2 V. At the system level, we demonstrate a spectral efficiency and a data rate of 6.6 b s^−1^ Hz^−1^ and 20 Mb s^−1^, respectively. A comparison between rigid and flexible samples shows absolute differences of only 0.2 b s^−1^ Hz^−1^ and 2.9 Mb s^−1^ for the spectral efficiency and the data rate, respectively. The presented results utilizing an industrially relevant printing technique such as inkjet printing will contribute to the future implementation of organic receivers into indoor VLC applications and provide the basis for incorporating OPDs into advanced multi-device systems such as IoT and optical wireless sensor networks.

## Results and discussion

### Design and fabrication

**Figure 1:**
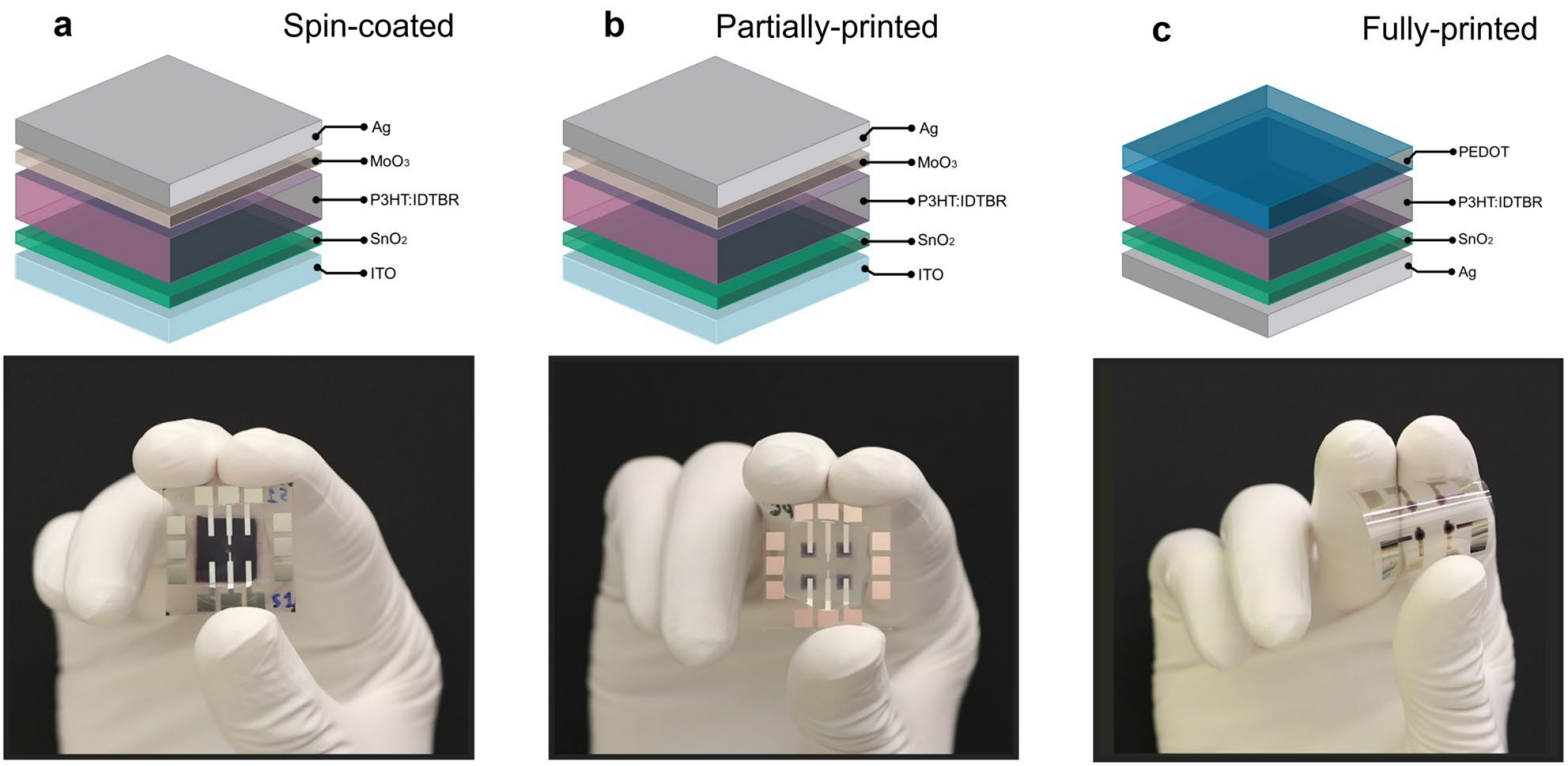
OPD architectures. (**a**) Material stack and layout of the spin-coated devices. (**b**) Material stack and layout of the partially-printed devices. In this case the P3HT:IDTBR active layer was inkjet-printed. (**c**) Material stack and layout of the fully-printed devices. In this case all materials were inkjet-printed.

Figure 1 shows the different architectures as well as the layouts employed in the fabricated OPDs. The device which fabrication was based on the spin-coating method is presented in Fig. 1a. This device was fabricated on an indium tin oxide (ITO) covered glass substrate. ITO was used as a transparent electrode onto which a SnO_2_ film used as a hole-blocking layer, and the bulk heterojunction (BHJ) active layer formed by the donor poly(3-hexylthiophene) (P3HT) and the small-molecule acceptor 5,5′-[[4,4,9,9-Tetraoctyl-4,9-dihydro-s-indaceno[1,2-b:5,6-b’]dithiophene-2,7-diyl]bis(2,1,3-benzothiadiazole-7,4-diylmethylidyne)]bis[3-ethyl-2-thioxo-4-thiazolidinone] (IDTBR) were fabricated by spin coating. A MoO_3_ film used as electron-blocking layer, and Ag used as the complementary electrode were vacuum evaporated. The pixel active area of this and all other employed devices is defined by the overlap between the electrodes and is equal to 1 mm^2^. Figure 1b corresponds to a partially-printed device. In this case, the P3HT:IDTBR active layer was inkjet-printed, while the rest of the stack remained the same as in the spin-coated sample. Reduced material consumption and spatial separation between devices can be readily seen when comparing the photograph of the fabricated device to that of the spin-coated sample. Figure 1c corresponds to a fully-printed device, which was fabricated on a poly(ethylene 2,6-naphthalate) (PEN) flexible substrate. In terms of architecture, the fully inkjet-printed device consists of Ag as the bottom electrode, SnO_2_ as the hole-blocking layer, P3HT:IDTBR as the BHJ active layer, and poly(3,4-ethylenedioxythiophene) polystyrene sulfonate (PEDOT:PSS) as the top transparent electrode. Due to their compatibility with flexible substrates and the use of industrially relevant printing techniques, the fabricated printed OPDs could potentially facilitate numerous flexible and wearable applications in a variety of fields. Herein, we investigate their suitability for indoor VLC systems and the change in performance that occurs when the fabrication process is changed from spin coating to inkjet printing.

### Characteristics of OPDs for different architectures

**Figure 2:**
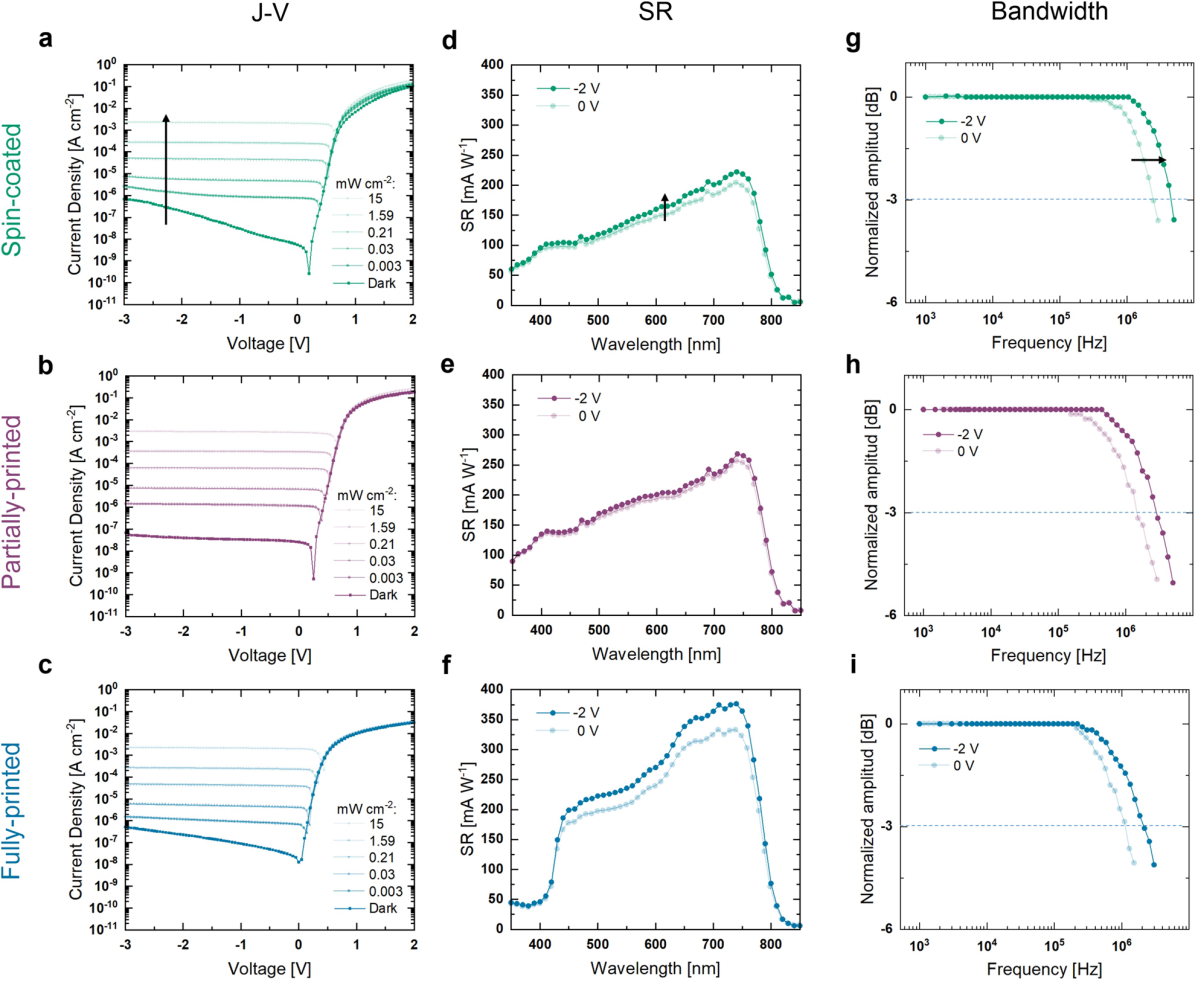
OPD characteristics for different architectures. (**a**–**c**) J-V characteristics of the three different architectures studied. A monochromatic light source (λ = 520 nm) was used for illumination. (**d**–**f**) Spectral responsivities of the three different architectures studied. (**g**–**i**) Cut-off frequency measurements of the three different architectures studied.

Figure 2 shows the J-V characteristics, spectral responsivity (SR), and 3_ dB_-bandwidth of the three different architectures being investigated. It is important to point out that, the ink formulations optimized for inkjet printing differ from those optimized for spin coating. Consequently, the active layers of the spin-coated samples shown here use chlorobenzene (CB) as their solvent system, (Fig. 1a), different from the active layers of printed samples using 1,2-Dichlorobenzene (DCB), (see Fig. 1b,c). This change in solvent composition is necessary for better compatibility with the printing process, particularly in order to avoid clogging of the printing nozzles. By taking advantage of the higher boiling-point of DCB as compared to CB, we achieve a lower evaporation rate and an optimized ink jetting from the printing cartridge. Figure 2a–c presents the J-V characteristics, where each device was measured under dark and monochromatic light (λ = 520 nm) conditions with the incident optical power reaching up to 15 mW cm^−2^. As it can be seen, the three architectures show a characteristic rectifying behavior with dark currents of 189, 39, and 227 nA cm^−2^ measured at  − 2 V for the spin-coated, partially-printed, and fully-printed cases, respectively. These values demonstrate a successful device stack for the different architectures employed. As expected, the current output of the devices increases with the illumination intensity. Supplementary Fig. S1a–c shows that, when biased at  − 2 V, the devices provide a good linear dynamic range (LDR) with values of 81, 97, and 79 dB for the spin-coated, partially-printed and fully-printed cases, respectively.

Figure 2d–f depicts the SR values at the bias voltage values of 0 and  − 2 V. The three architectures exhibit a broad photoresponse, characteristic of their P3HT:IDTBR active layer, covering the complete visible range and extending into the near-infrared (NIR). The current response of the spin-coated and the partially-printed samples is consistent across the entire spectral range, exhibiting a steady increase in the response from 400 nm to their peak value at 740 nm, followed by a gradual decrease until 850 nm. In terms of the responsivity of the devices, the change in solvent system did not have a strong effect as seen in Fig. 2d and e. The detailed investigation of the BHJ morphological differences of the two ink formulations and relation to the figures-of-merit are out of the scope of this study. However, they should be addressed in future work as they are fundamental to the optimization of OPD-based VLC systems. The fully-printed device shows a more abrupt increase in responsivity with a shoulder-like shape between 400 and 600 nm. The fully-printed device also yields higher overall photoresponse performance. This change in SR can be attributed to the different active layer thicknesses in the samples. While the spin-coated and partially printed devices have a similar active layer thickness of ~ 220 and ~ 240 nm; the fully-printed devices have an increased thickness of ~ 350 nm. A thicker active layer allows for higher light absorption and can lead to an increase in photoresponse. In addition, they can also lead to changes in the shape of their SR profiles. This can be further appreciated in Supplementary Fig. S2, which shows the SR of the fully-printed sample as compared to another fully-printed sample of thinner (~ 180 nm) active layer thickness. The fully-printed sample with decreased thickness shows a similar spectral shape to the partially-printed and spin-coated samples. The change in SR suggests that even moderate adjustments in active layer thickness can significantly affect the SR. These findings are in agreement with previous literature investigating the effects of varying BHJ thickness^39–42^. The performance of fully-printed devices with thinner active layers was lower compared to the devices with thicker active layers, therefore, devices with thicker active layers were employed in further characterizations. In general, all devices reach responsivities above 200 mA W^−1^ with the fully-printed architecture achieving a maximum SR value of 376 mA W^−1^. The peak responsivity of the spin-coated sample is 222 mA W^−1^ (at 740 nm,  − 2 V), and the peak responsivity of the partially-printed sample is 268 mA W^−1^ (at 740 nm,  − 2 V). These SR values are similar to other values reported for state-of-the-art devices^7,8,12,43–50^.

Figure 2g–i shows the frequency response of the fabricated OPD devices. The OPDs reached  − 3_ dB_ cut-off frequency bandwidths (ƒ_−3 dB_) between 2 and 5 MHz at a bias voltage of  − 2 V. The spin-coated architecture achieved the maximum value with a ƒ_−3 dB_ of 4.4 MHz. In contrast, the partially-printed samples on glass show a ƒ_−3 dB_ of 2.8 MHz. As a comparison, we investigated the bandwidth of a reference spin-coated sample containing an active layer with the same solvent system (DCB) as the printed devices (see Supplementary Fig. S3). It can be seen that both samples achieve a similar performance with a ƒ_−3 dB_ ~ 2.8 MHz at  − 2 V. This result suggests that the fabrication process can be switched from spin-coating to printing while maintaining the bandwidth of the device. However, the variation in performance shown from Fig. 2g–h originates from the different processing of the active layers such as the ink formulation. Previous studies have shown that a change of solvent from CB to the higher boiling point DCB can directly influence the domain size and crystallinity in P3HT-based blends^51,52^, including P3HT:IDTBR BHJs^53^. Changes in our ink formulations can, therefore, influence the exciton diffusion and the charge separation efficiency via these mechanisms, directly affecting the performance of the device. Furthermore, the mechanically-flexible samples reached a maximum ƒ_−3 dB_ of 2.1 MHz at  − 2 V, demonstrating a high device performance even for fully-printed, flexible samples. The lower ƒ_−3 dB_ is attributed to the higher active layer thickness in the fully-printed device as compared to the partially-printed device. This is supported by previous results from our group showing that a P3HT:IDTBR active layer thickness of ~ 200 nm represents a well-balanced trade-off between the transit- and RC-limit for our devices, and showing that a further increase in the active layer thickness leads to an increase in the transit time and thus to a drop in ƒ_−3 dB_^6^. In general, the reported ƒ_−3 dB_ values are among the highest values reported for OPDs^5,30,31^, and are higher than those of other OPDs previously tested in VLC systems^32–37,54^.

### OPDs-based VLC

**Figure 3:**
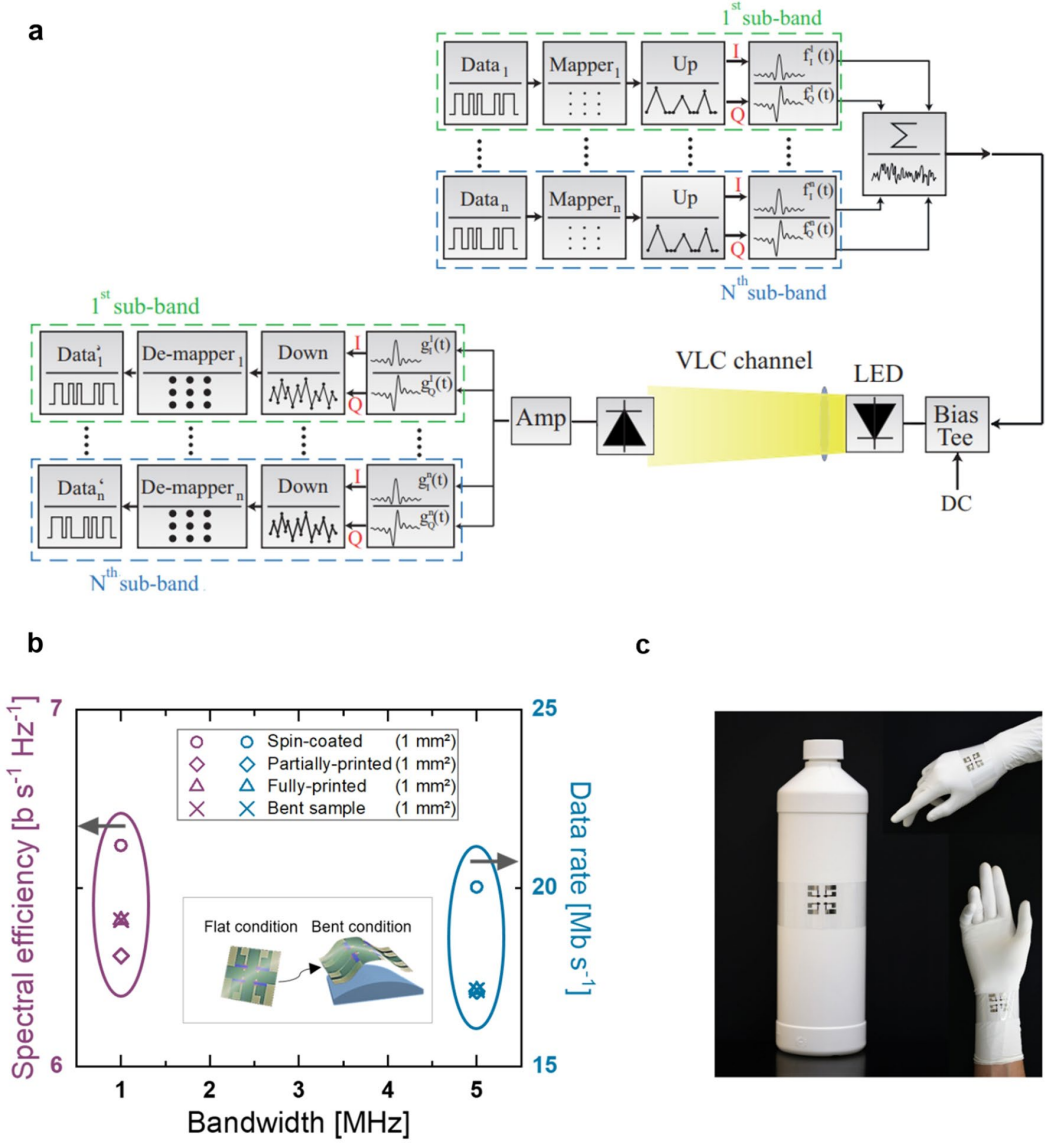
Integration of OPDs within VLC system. (**a**) Schematic block diagram of the VLC system. In the presented system, the three different OPD architectures were individually tested as receivers. (**b**) Measurements of spectral efficiency and data rate for the three different OPD architectures. The fully printed samples were tested both in rigid and mechanically flexed scenarios. The inset shows a schematic of the sample under flat and bent conditions. The bending radius on the test structure used for bending was set to 50 mm. (**c**) Photograph of the fully-printed and flexible OPD samples conforming to different curved surfaces.

Figure 3a shows a schematic block diagram of the VLC system being employed. Here, we have adopted the multi carrierless amplitude and phase (*m*-CAP) modulation format, and its generation is outlined in the literature^55^. First, the *m*-CAP signal generated in Matlab was uploaded to an arbitrary waveform generator, the output of which is used for intensity modulation of the LED (an OSRAM LW W5SM with a bandwidth of 1.2 MHz) via the bias-tee module. The spectrum of the LED, which matches the OPD responsivity range, can be seen in Supplementary Fig. S4. Moreover, a focal lens was used at the transmitter to increase the optical power level at the receiver (i.e. higher signal-to-noise ratio (SNR)), resulting in an intensity of 21 mW cm^−2^. At the receiver, the different OPD architectures, namely, spin-coated, partially-printed, and fully-printed, were tested individually. A transimpedance amplifier (Thorlabs AMP140) was used to boost the regenerated *m*-CAP signal level. The captured signal using a real-time oscilloscope was processed in the Matlab domain in order to regenerate the estimated data stream for comparison with the transmitted data sequence to determine the bit error rate (BER).

Figure 3b displays the results of the VLC measurements for the different OPD architectures. The figure shows the maximum system data rate and spectral efficiency of the described OPDs. The corresponding spectrums received during measurement can be seen in Supplementary Fig. S5. Note, all measurements were performed with the OPD biased at  − 2 V and meeting the target 7% forward error correction BER limit. Moreover, in our VLC-system we are limited by the 3_ dB_ bandwidth of the LED and the photodetectors. As mentioned above, the data in Fig. 3b was obtained by transmitting an m-CAP signal through the LED + OPD channel (see Fig. 3a). This modulation format allows us to divide the occupied bandwidth into subbands that are affected by the distortion independently and load a different amount of data into each subband allowing us to effectively use a higher bandwidth beyond the 3_ dB_ bandwidth of our devices. The maximum spectral efficiency and the maximum data rate were measured at 1 and 5 MHz, respectively. In both figures of merit, we observed an equivalent operation for the different devices. While the spectral efficiency for the spin-coated OPD was the highest at 6.6 b s^−1^ Hz^−1^, this corresponds to only 0.3 and 0.2 b s^−1^ Hz^−1^ absolute difference with respect to the partially- and fully-printed OPDs, respectively. For the obtained data rates, we observed a similar outcome with an absolute difference of only 2.9 Mb s^−1^ between the spin-coated and printed samples. In this case, the maximum data rate corresponds to 20 Mb s^−1^. The achieved data rates are sufficient for several applications including (i) indoor IoT for identification and promotion information, control signals, and indoor localization^21^, (ii) intelligent transportation systems^22^, and (iii) E-health such as communication between on-body sensors to IoT nodes^23^. Overall, the results show that all OPD devices exhibit properties that are suitable for VLC systems with performance comparable to other advanced OPDs used in VLC systems^19,32–38^. Most importantly, we have demonstrated that the developed OPDs maintain their performance in the VLC system even after adopting the fabrication method based on the fully-inkjet-printed process deposited on a flexible substrate. This is noteworthy because the printed samples possess bending capabilities and manufacturing advantages that may be exploited for a variety of new applications such as IoT technologies, wearable and on-body sensors. In this regard, we also tested the performance of the OPDs under mechanical stress by measuring both the spectral efficiency and data rate at different bending radiuses of the receiver substrate. As shown in Fig. 3b, the fully printed OPDs were able to maintain their performance under the bending conditions. The inset in Fig. 3b shows a schematic of the bending conditions, where a semi-cylindrical platform with a bending radius of 50 mm was used for bending the sample. A bending radius of 100 mm yielded the same results maintaining the achievable spectral efficiency and data rate. Incompatible with the OPD-encapsulation method (i.e. lack of mechanical flexibility of the encapsulation glue), no tests were carried out in the VLC system for bending radiuses below 50 mm. Nevertheless, as depicted in Fig. 3c, a device with a bending radius of ≥ 50 mm can have several useful applications as it resembles the positioning of the sample over many curved surfaces such as bottles or tubes, as well as the hand or forearm of a person. For complementarity, we characterized the SR of the flexible samples at radiuses of 100, 50, 10 and 5 mm. These measurements were performed outside the VLC system and inside a nitrogen-filled glovebox, where we measured the generated photocurrent of OPDs without encapsulation and under monochromatic light (λ = 520 nm) at an intensity of 10 mW cm^−2^. The results displayed in Supplementary Fig. S6 show that our devices are capable of maintaining a high and consistent photoresponse despite exposure to the increased bending conditions.

Table 1 summarizes the VLC and SR measurements for the OPD samples under different bending conditions. In terms of spectral response, we observed a maximum absolute difference of only 17 mA W^−1^ when comparing the flexible sample under bent and flat conditions. Thus, we expect that with enhanced encapsulation compatibility of the OPDs used in the VLC system, the devices could also maintain a consistent data rate and spectral efficiency performance even under the increased bending conditions that we tested in terms of photoresponse. Although modifications to the encapsulation method may be necessary for radiuses below 50 mm, radiuses of ≥ 50 mm remain highly favorable as compared to rigid samples. Thus, we consider the presented OPD devices to be a promising option in IoT applications with uneven and curved surfaces.

**Table 1:** Summary of VLC and SR measurements performed on the different OPD samples.

Fabrication	Spin-coated	Partially-printed	Fully-printed				
Bending radius (mm)	Flat	Flat	Flat	100	50	10	5
SR (mA W^−1^) @ 0 V, λ = 520 nm	115	168	181	170	168	164	175
Spectral efficiency (b s^−1^ Hz^−1^) @ − 2 V	6.62	6.31	6.41	6.41	6.41	–	–
Data rate (Mb s^−1^) @ − 2 V	20.0	17.1	17.1	17.1	17.1	–	–

## Conclusion

In summary, we have, for the first time, demonstrated the successful integration of high-performance OPDs with different architectures into an indoor VLC system. The presented devices offer improved performance compared with other OPDs previously reported in literature. We reported, ƒ_−3 dB_ values between 2 and 5 MHz at  − 2 V and visible-range SR values above 200 mA W^−1^ for all architectures. By switching the fabrication process, we showed that the performance of the developed OPDs in a VLC link can be maintained despite shifting the fabrication method to a fully-inkjet-printed process deposited on a mechanically flexible substrate. We showed that the absolute difference in the spectral efficiency is only 0.2 b s^−1^ Hz^−1^ between rigid and flexible samples. With the bending capabilities, fast detection speeds, and manufacturing advantages of the printed samples, we expect these devices to be used in IoT applications with curved surfaces as part of VLC systems.

Future work will aim to explore new encapsulation methods for the developed OPDs oriented towards high mechanical flexibility in order to further facilitate their implementation into new wearable applications and into other advanced and complex systems compatible with IoT.

## Methods

### OPD fabrication

The fabrication of the devices via spin coating and inkjet printing was performed in air inside a cleanroom environment. Only the preparation of active layer solutions and the annealing of the deposited active layers was performed inside a glovebox with nitrogen environment. For encapsulation, devices were covered with a curable adhesive. The processing of the spin-coated and partially-printed OPDs took place over glass substrates containing pre-structured ITO electrodes. SnO_2_ layers (25 nm) were spin-coated on top using a solution of commercial SnO_2_ nanoparticle ink (Avantama N-31). Subsequently, the samples were annealed at 120 °C for 5 min. Independent solutions of P3HT (RIEKE Metals) and the small-molecular IDTBR (1-MATERIALS) were prepared in CB (40 mg/mL) for the spin-coated devices or DCB (20 mg/mL) for the partially-printed devices and then mixed in a 1:1 ratio to form the BHJ. Thereafter, the active layers were either spin-coated (220 nm), or inkjet-printed (240 nm) followed by an annealing step at 140 °C for 10 min. Afterwards, MoO_3_ (30 nm) and silver (100 nm) layers were thermally evaporated on top to complete the devices.

The processing of the fully-printed OPDs took place over PEN foils by using a Dimatix printer (DMP 2831), with 10 pL 16 nozzle Fujifilm Dimatix cartridges. In this case, an Argon plasma treatment was performed on the PEN substrates for one minute in order to adjust the surface energy prior to the OPD fabrication. Afterwards, Ag bottom electrodes (100 nm) were printed using a 30–35 wt% Ag nanoparticle ink (Sigma-Aldrich TGME Silver Dispersion) and annealed at 120 °C for 10 min. The SnO_2_ layers were then printed on top of the Ag electrodes with a modified solution using the commercial nanoparticle ink combined with diethylene glycol (DEG) in a 2:1 mixture. After this, the samples were annealed at 120 °C for 5 min. The BHJ solution was formed through preparation of independent solutions of P3HT (RIEKE Metals) and IDTBR (1-MATERIALS) in DCB (20 mg/mL) that were stirred overnight and then mixed together (1:1). The resulting solution was inkjet-printed (350 nm) in air, followed by an annealing step inside a N_2_-filled glovebox at 140 °C for 10 min. Finally, a transparent electrode (300 nm) based on PEDOT:PSS (Clevios FHC-Solar Heraeus) with 0.3 vol% of Zonyl FS-300 (Fluka analytical) was inkjet printed as top electrode and annealed at 120 °C for 5 min to complete the OPD stack. Filtering of all solutions was done using 0.45 µm polyvinylidene fluoride (PVDF) filters. The inkjet-printing waveforms were designed independently for each layer. To determine the thickness of the spin-coated and printed layers we employed a surface profilometer (Veeco, Dektak 150).

### OPD characterization at device level

To measure the current–voltage (I–V) characteristics we utilized an Agilent 4155 C semiconductor parameter analyzer. I-V measurements under illumination were performed using an LED light source with a wavelength of λ = 520 nm. The LED was powered with a Keithley 2636 A source measure unit (SMU). The light intensity was adjusted using neutral density (ND) filters (Thorlabs NDUVxxA/NE5xxB).

To perform SR measurements a monochromator (Acton SP-2150i) was used to selectively filter light from a 450 W OSRAM XBO Xenon discharge lamp. The light was modulated at a frequency of 173 Hz with a chopper wheel, and the OPD signal was amplified using a Femto DHPCA-100 amplifier. An SR830 lock-in amplifier was used to measure the output signal.

To characterize the bandwidth, we varied the frequency of a square-light signal and measured the transient current under illumination. The light was modulated using an Agilent 33,522 A function generator. As a light source, we used an Oxxius LBX520 laser. The signal was recorded using an Agilent DSO 6102 A oscilloscope.

### Characterization of OPDs in VLC link

For the VLC with OPD-based receiver, we generated the data stream and converted into the *m*-CAP in the Matlab domain and then loaded it into an arbitrary function generator (Teledyne LeCroy T3AWG3252) to generate the electrical version of the *m*-CAP signal. The *m*-CAP signal was used for intensity modulation of the LED (OSRAM LW W5SM, 1.2 MHz) via a bias tee (Mini-Circuits BIAS-TEE ZFBT-4R2GW). A focusing lens was used at the transmitter in order to increase the optical power level at the receiver. To bias the OPDs during the measurements we utilized a SMU. The output of the OPD was amplified using a transimpidance amplifier (Thorlabs AMP140) with the gain and MHz bandwidth of 10 kV A^−1^ and 10 MHz, respectively, the output of which was captured using a real-time oscilloscope (Keysight DSO0104A) for further processing in the Matlab domain. After down-sampling and demodulation, we compared the recovered M-ary quadrature amplitude modulation (M-QAM) symbols with the transmitted data for BER estimation. We used a BER that corresponds to the limit of 7% forward error correction. To improve the system throughput, we used a pilot binary phase-shift keying (BPSK) signal to load an appropriate k-value to individual subcarriers based on the measured SNR. As an optimization technique, we used a β-optimization as described in^56^.

### Supplementary Information

Supplementary Information.

## Data Availability

The data that supports the findings of this study are available from the corresponding author upon reasonable request.
