# Supplementary material for: Inkjet-printed high-performance and mechanically flexible organic photodiodes for optical wireless communication
Source: Sci Rep. 2024 Feb 8;14:3296. doi: 10.1038/s41598-024-53796-5 (PMC10853278; doi:10.1038/s41598-024-53796-5)
Supplement: Supplementary file 1 — Supplementary Information. [file 41598_2024_53796_MOESM1_ESM.pdf]

Supporting information

Inkjet-printed High-performance and Mechanically Flexible Organic Photodiodes for Optical Wireless Communication

Luis Arturo Ruiz-Preciado[1,2], Petr Pesek[3], Carlos Guerra-Yánez[3], Zabih Ghassemlooy[4], Stanislav Zvánovec*[3], and Gerardo Hernandez-Sosa*[1,2,5]

[1] Light Technology Institute, Karlsruhe Institute of Technology, Engesserstr. 13, 76131 Karlsruhe, Germany

[2] InnovationLab, Speyererstr. 4, 69115 Heidelberg, Germany

[3] Faculty of Electrical Engineering, Czech Technical University in Prague, Dejvice-Praha 6, Prague, 16627, Czech Republic

[4] Optical Communications Research Group, Faculty of Engineering and Environment, Northumbria University, Newcastle, UK

[5] Institute of Microstructure Technology, Karlsruhe Institute of Technology, Hermann-von-Helmholtz-Platz 1, 76344 Eggenstein-Leopoldshafen, Germany

* corresponding author(s): xzvanove@fel.cvut.cz; gerardo.sosa@kit.edu

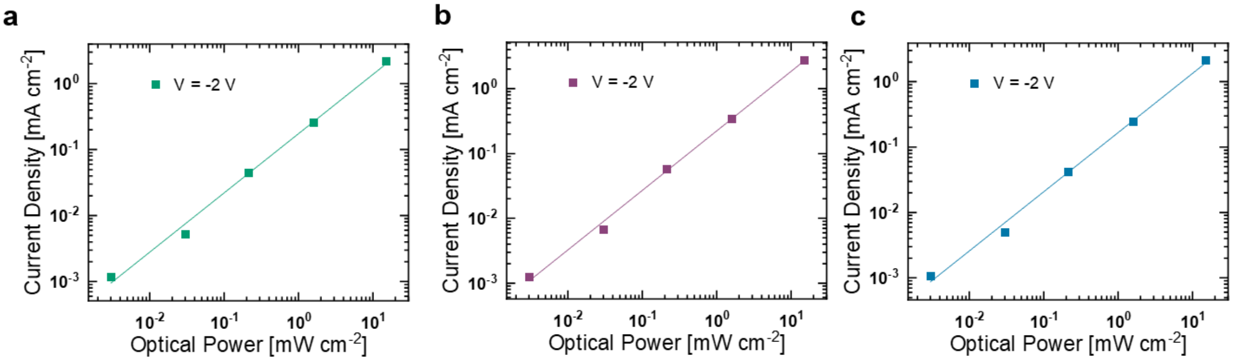

Supplementary Figure S1– LDR of the different architectures

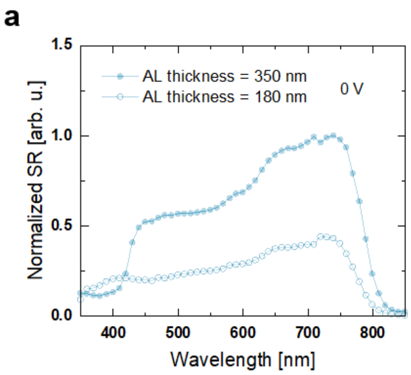

Supplementary Figure S2– SR of fully-printed devices with different AL thickness

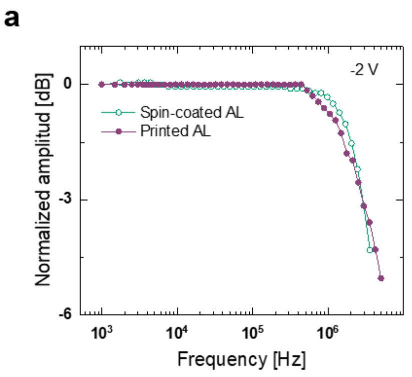

Supplementary Figure S3– Bandwidth measurements of spin-coated and printed samples using the same solvent system

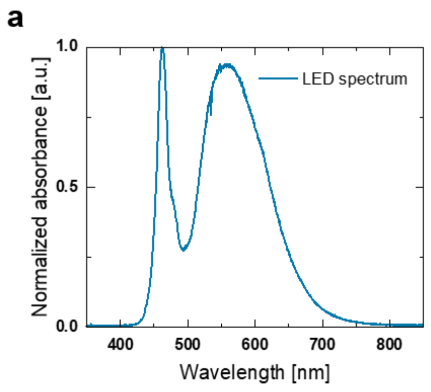

Supplementary Figure S4– Spectrum of LED used as transmitter

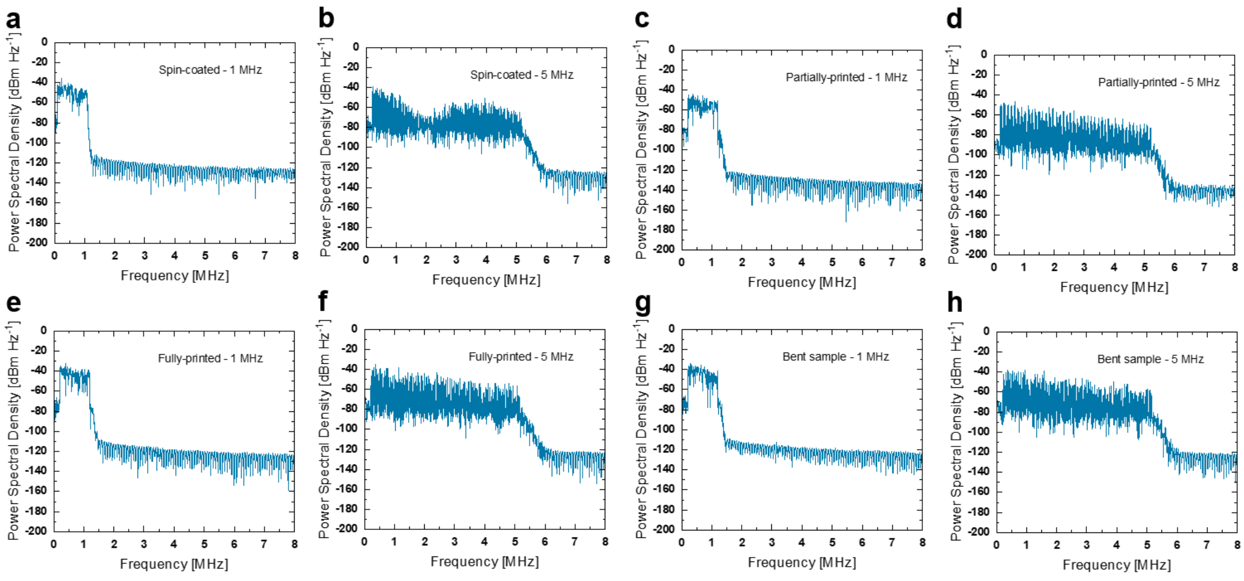

Supplementary Figure S5– Spectrums received during VLC measurements

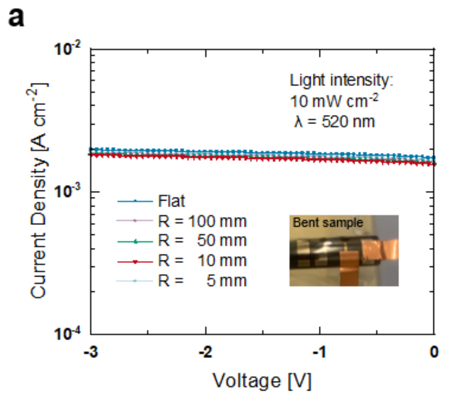

Supplementary Figure S6– Output current of OPDs measured during bending under monochromatic light
